# A Systematic Evaluation of Feature Encoding Techniques for Gait Analysis Using Multimodal Sensory Data

**DOI:** 10.3390/s24010075

**Published:** 2023-12-22

**Authors:** Rimsha Fatima, Muhammad Hassan Khan, Muhammad Adeel Nisar, Rafał Doniec, Muhammad Shahid Farid, Marcin Grzegorzek

**Affiliations:** 1 Department of Computer Science, University of the Punjab, Lahore 54590, Pakistanshahid@pucit.edu.pk (M.S.F.); 2 Department of Information Technology, University of the Punjab, Lahore 54000, Pakistan; adeel.nisar@pucit.edu.pk; 3 Faculty of Biomedical Engineering, The Silesian University of Technology, 44-100 Gliwice, Poland; rafal.doniec@polsl.pl; 4 Institute of Medical Informatics, University of Lübeck, 23562 Lübeck, Germany

**Keywords:** gait analysis, human activity recognition, time series sensory data, feature encoding, classification

## Abstract

This paper addresses the problem of feature encoding for gait analysis using multimodal time series sensory data. In recent years, the dramatic increase in the use of numerous sensors, e.g., inertial measurement unit (IMU), in our daily wearable devices has gained the interest of the research community to collect kinematic and kinetic data to analyze the gait. The most crucial step for gait analysis is to find the set of appropriate features from continuous time series data to accurately represent human locomotion. This paper presents a systematic assessment of numerous feature extraction techniques. In particular, three different feature encoding techniques are presented to encode multimodal time series sensory data. In the first technique, we utilized eighteen different handcrafted features which are extracted directly from the raw sensory data. The second technique follows the Bag-of-Visual-Words model; the raw sensory data are encoded using a pre-computed codebook and a locality-constrained linear encoding (LLC)-based feature encoding technique. We evaluated two different machine learning algorithms to assess the effectiveness of the proposed features in the encoding of raw sensory data. In the third feature encoding technique, we proposed two end-to-end deep learning models to automatically extract the features from raw sensory data. A thorough experimental evaluation is conducted on four large sensory datasets and their outcomes are compared. A comparison of the recognition results with current state-of-the-art methods demonstrates the computational efficiency and high efficacy of the proposed feature encoding method. The robustness of the proposed feature encoding technique is also evaluated to recognize human daily activities. Additionally, this paper also presents a new dataset consisting of the gait patterns of 42 individuals, gathered using IMU sensors.

## 1. Introduction

Gait refers to the movement patterns of an individual’s walk. It encompasses the rhythm, speed, and style of movement which require a strong coordination of the upper and lower limbs. The process of gait analysis involves evaluating an individual’s walking pattern to assess their biomechanics and identify any abnormalities or inefficiencies in their movement [1]. It has been an active research area over the last few years due to its utilization in numerous real-world applications, e.g., clinical assessment and rehabilitation, robotics, gaming, entertainment, etc. [2,3]. The quantitative gait analysis has also been explored as a biometric modality to identify a person [4]. The gait analysis has several advantages over other existing modalities; in particular, it is unobtrusive and difficult to steal or falsify. Gait analysis is a challenging task because it involves the complex coordination of human skeletal, muscular, and nervous systems. Additionally, the gait can be affected by a wide range of factors, such as age, injury, disease, and environmental conditions. This results in intra-personal variations, which are always greater than inter-personal variations.

**Figure 1:**
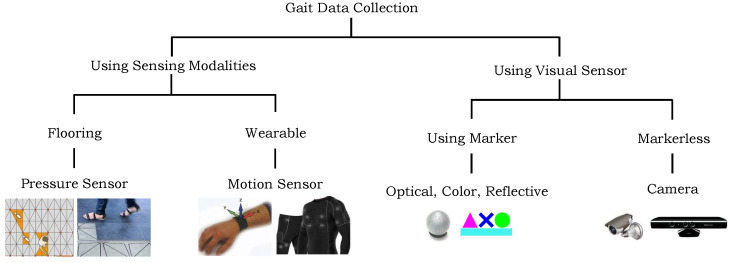
A set of sensing modalities that can be used for gait data collection.

The gait data can be gathered via a variety of sensing modalities that can be broadly divided into two groups: using sensing modalities [5] and using visual cameras [3]. Figure 1 illustrates these sensing modalities that can be used for data gathering. Each of these modalities have their own strengths and limitations, and researchers choose to use single or a combination of several modalities to capture gait data based on their research questions and goals. Vision-based systems have been extensively used in the gait analysis due to their higher precisions however, their use raises confidentiality and privacy concerns [6]. Conversely, the digital sensors such as inertial measurement units (IMU), and pressure sensors have been also used to collect the gait data. These tiny sensors can easily be embedded in our environment, including walking floor, wearable devices, and clothes. Furthermore, a few of such sensors are already embedded in our daily used digital gadgets, e.g., smart phones, smart watches, and smart glasses [7]. These devices generate data in the form of pressure signals, velocity, acceleration, and positions which can be used to represent the gait.

Analyzing gait requires the use of sophisticated tools and techniques. Since the gait comprises continuous time series data, we need to extract a set of relevant features from these raw data to represent gait patterns. There are several approaches in the literature to extract features, and they can be categorized into three groups: (1) handcrafted [8], (2) codebook-based [9], and (3) automatic deep learning-based [10] feature extraction approaches. The handcrafted features are usually a few statistical quantities, e.g., mean, variance, skewness, kurtosis, etc., that are extracted from raw data based on the prior knowledge of experts in the application domain. Later, these features are fed to machine learning algorithms for classification. Typically, they are simple to implement and are computationally efficient; however, they are designed to solve a specific problem and are not robust [7]. The codebook-based feature learning techniques follow the channel of Bag-of-Visual-Words (BOVWs) approach [11] to compute gait features. Specifically, they employ clustering algorithms, e.g., k-means, to build a dictionary (also known as codebook) using the gait sub-sequences from raw data. The data are grouped based on their underlying similarities, and the clusters’ centers are known as “codewords”. Later, a histogram-based representation is computed for other sequences by tying them to the closest-related codeword. Codebook-based techniques are proven to be robust as they can capture more complex patterns in the gait data; however, they are computationally expensive [12]. Deep learning-based techniques automatically compute discriminative features directly from the input data using artificial neural networks (ANNs). A deep network usually comprises several layers where each layer consists of artificial neurons. After obtaining input, a specific feature map is computed by the neurons in each layer and then forwarded to the next layer for further processing, and so forth. Finally, the network’s last layers generate highly abstract feature representations of the raw sensory data. A few examples of deep learning approaches for gait analysis are convolutional neural networks (CNNs) [13], recurrent neural networks (RNNs) [14], and long short-term memory networks (LSTMs) [15]. Deep learning-based approaches can automatically learn complex features from raw data and are adaptable to new datasets and scenarios; however, they are computationally much more expensive and require a huge amount of labeled instances to optimally choose the hyperparameters’ values of the deep network. The entire process is greatly hampered by the lack of prior information necessary to encode the appropriate features in the application domain and the choice of the best parameters for the machine learning algorithms [7].

This paper presents a systematic evaluation of numerous feature learning techniques to encode multimodal time series sensory data for gait analysis. Furthermore, the proposed feature encoding techniques also explored to recognize the human daily activities which were recorded using sensory data too.Specifically, we presented three different encoding techniques to recognize the walking styles. In the first technique of handcrafted features, we computed eighteen different statistical quantities from the raw sensory data, and they are fused together in a single feature vector to obtain the high-level representation. In the second feature learning technique, we build a codebook using k-means clustering algorithm, and the high-level feature representation is obtained using Locality-constraint linear encoding (LLC) [16]. In both of the aforementioned techniques, we explored the effectiveness of Support Vector Machines (SVMs) and Random Forest (RF) as machine learning algorithms to recognize different walking styles. Third, we presented two deep learning models, a CNN and an LSTM, which employed raw sensory data as input to recognize walking sequences. The effectiveness of the proposed features was assessed on four datasets, namely Gait-IMU, MHEALTH [17], WISDM [18], and UCI-HAR [19]. An extensive experimental evaluation of different feature encoding techniques was carried out along with different values of hyperparameters, and the recognition scores were compared with recent state-of-the-art approaches. The proposed framework establishes a solid foundation and generic framework to encode any time series multimodal sensory data. We believe that the raw time series sensory data of other sensing modalities such as SensFloor, skin temperature sensors, and electrocardiograms (ECGs) can also be encoded using the proposed approaches to perform recognition tasks. The proposed framework can be employed in numerous emerging fields and technologies, including healthcare and rehabilitation, sports, assistive living, and others. The major contributions of the proposed manuscript are as follows:

- A comparative study of three different feature encoding techniques is presented.
- A comprehensive review of the existing techniques is presented, and their benefits and drawbacks are discussed.
- The effectiveness of various machine learning methods is evaluated in order to classify the multimodal sensory data.
- The computational analysis of different feature encoding techniques is presented.
- The robustness of the proposed technique is assessed on several applications, including walking styles, human activities, etc.
- A rigorous evaluation of all the feature encoding techniques is carried out on four large datasets.
- A large gait dataset that is collected using IMU sensors is proposed.

## 2. Related Work

**Figure 2:**
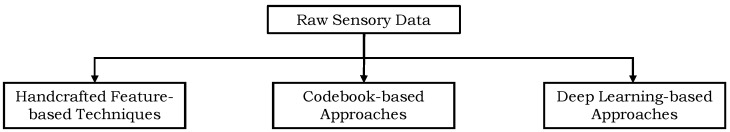
Classification of existing feature encoding techniques using sensory data based on their underlying computing methods.

Gait analysis is a fundamental tool in biomechanics that allows for both quantitative and qualitative assessment of human movement. It involves the analysis of the spatiotemporal parameters, kinematics, and kinetics of gait, which are important indicators of the locomotion function [20,21,22]. This paper mainly emphasizes on gait analysis using multimodal time series sensory data. The existing techniques employed either a pressure sensor [23,24,25] or an IMU [26,27] to encode walking patterns. In recent years, a large number of algorithms has been presented to investigate the movement of human body parts for clinical and behavioural assessment. They can be broadly divided into three groups, as depicted in Figure 2. In the following, a brief summary of a few techniques from each group is presented.

### 2.1. Handcrafted Feature-Based Techniques

These set of techniques either compute several statistical measurements on input data (e.g., average, variance, skewness) or extract more complex gait characteristics, which may include stride length, joint angles, and other related features. In the context of machine learning, handcrafted features refer to manually designed features that are extracted from raw data and used as input to a classifier [8,28,29,30]. For instance, the technique proposed in [31] extracted several statistical quantities on input data (e.g., mean, median, mode, standard deviation, skewness, and kurtosis) to show the gait fluctuation in a patient with Parkinson’s. They employed Fisher Discriminant Ratio to determine the most discriminatory statistical feature. A comparative study of different handcrafted features is proposed in [28] for the early detection of traumatic brain injury. The authors employed the location and accelerometer sensory data of a smartphone to extract nine gait features, including coefficient of variance, step count, cadence, regularity in step, stride, etc. Similarly, the authors of [32] extracted standard deviation, skewness, kurtosis, and bandwidth frequency features from the accelerometer data of an IMU sensor mounted on the subject’s lower back to distinguish between normal and stroke gait patterns. The study presented in [33] extracted thirty-eight statistical quantities, including maximum, minimum, average, spectral energy, etc., to monitor and quantify various human physical activities using a smartphone’s IMU sensory data. Similarly, the technique proposed in [34] employed frequency domain features to assess gait accelerometer signals. The approach proposed in [35] analyzed the gait sensory data using adaptive one-dimensional time invariant features.

These techniques appear simple in implementation; however, they are highly based on expert knowledge in the application domain. Additionally, they are designed to solve a specific problem and are not robust.

### 2.2. Codebook-Based Approaches

These techniques follow the work-flow of the BOVWs approach to encode the raw sensory input data into its compact but high-level representation. In particular, the input data are clustered based on similar patterns using a clustering algorithm (e.g., k-means) to create a dictionary. The cluster centers in the dictionary are known as codewords which describe the underlying variability in the gait data. Later, a final representation of the raw gait input data is achieved by estimating the distribution of these codewords in the gait sequence. This results in histogram-like features (where a histogram bin represents the occurrences of a specific codeword) which are fed to a classification algorithm for further analysis. For instance, the authors of [7,36] employed sensory information from commonly used wearable devices to identify human activities. They built a codebook using a k-means clustering algorithm, and the final gait representation is achieved using a simple histogram-binning approach. Similarly, the technique proposed in [37] employed IMU sensory data from smartphones and smartwatches to recognize Parkinson’s tremor using a codebook-based approach. In [38], research was carried out to recognize the human gait phase using two sensors: an accelerometer and a gyroscope. A codebook-based approach was explored to extract gait features from the raw sensory data. Similarly, the authors of [39] presented an approach to determine the best working positions for various movement phases and to guide the performer on how to keep them while performing physical activities. They explored a k-means clustering codebook to encode the different working postures for each phase of movement. In [40], a separate codebook is constructed to each sensor modality, including accelerometer, gyroscope, and magnetometer. The resulting features were concatenated to form a compact and high-level feature vector to classify human daily activities using SVM with a radial basis function (RBF) kernel. The technique in [41] presented a visual inertial system to recognize daily activities using two RGB-D (red, green, blue, and depth) detectors with a wearable inertial movement unit. They employed codebook-based technique on different sensing modalities to compute the desired features. In [12], a detailed comparison of different codebook-based feature encoding approaches is presented to recognize gait.

These techniques are proven to be robust, to some extent, as they can capture more complex patterns in the gait data; however, they are computationally expensive [12]. Additionally, this process of codebook computation may need to be performed again if new classes are added in the dataset [42].

### 2.3. Deep Learning-Based Approaches

Lately, automatic deep learning-based feature extraction approaches have been largely explored in different classification tasks due to their robustness in automatic feature extraction, generalization capabilities, and convincing recognition results. A deep network is typically an end-to-end architecture that can learn complicated patterns and relationships in input data using a fully automated feature extraction process. In particular, it comprises many layers of interconnected neurons. The network’s intricate and iterative structure allows for the learning of high-level features from input data as it passes it through (i.e., the weight adjustments of neurons and back-propagation of errors [43]). Numerous automatic feature learning-based techniques on gait analysis have been proposed in the past; convolutional neural networks (CNNs) [44] and long short-term memory (LSTM) [45] are a few examples to quote. For instance, the authors of [44] presented an IMU-based spectrogram technique to categorize the gait characteristics using a deep CNN. The authors of [46] explored a CNN to extract the appropriate high-level feature representation from pre-processed time series input data. They turned the input data into two-dimensional (2D) images where the y-axis represents various features and the x-axis represents the time.

Since a CNN is typically designed to process imagery data, a few other networks have also been explored to analyze gait [47,48,49]. For instance, the authors of [45] employed window-based data segmentation to classify gait sequences using a multi-model long short-term memory (MM-LSTM) network. To compute features from each window, the MM-LSTM network accepts input gait data that have been segmented into several overlapping windows of equal length. The authors of [50] employed recurrent neural networks (RNNs) for gait analysis. The method for calculating gait mechanics using an artificial neural network (ANN) with measured and simulated inertial measurement unit (IMU) data is suggested in [51]. The authors concluded that more precise estimations of gait mechanics can be obtained by combining the ANN with both simulated and actual data. In [52], a comprehensive overview of different deep learning models is presented to monitor the human gait.

The studies show that deep learning-based feature encoding techniques are effective tools for analyzing gait and have the potential to explore the underlying mechanics of gait. They provide automatic end-to-end learning; however, they also require complex computational resources and a huge amount of training data.

## 3. Overview of the Proposed Method

This paper presents a systematic evaluation of three different feature learning techniques to recognize the different walking styles using sensory data. The gait sequences were recorded using a set of IMU sensors. In the first method, we present eighteen different handcrafted features to represent a gait sequence. Secondly, we built a codebook using a k-means clustering algorithm, and the high-level gait features are obtained using locality-constrained linear coding (LLC). We evaluated two machine learning algorithms, namely SVM and RF, to validate the effectiveness of the aforementioned proposed features. Third, we employed two deep neural networks, namely CNN and LSTM, to automatically compute the discriminative gait features from raw sensory data. The proposed feature learning techniques were evaluated on four large datasets, and their results were compared with existing state-of-the-art techniques.

## 4. Proposed Feature Learning Techniques

This paper aims to present a comparative study of several feature extraction approaches to encode multimodal time series data for gait analysis. This section presents three different feature learning techniques to recognize walk styles using IMU sensory data. In the following, we briefly describe each technique.

### 4.1. Handcrafted Features

These features are computed either using a few simple statistical quantities (e.g., variance, skewness, etc.) or complex frequency domain-based features directly from raw sensory data [53]. We computed eighteen different handcrafted features and fused them together (via simple concatenation) to form a single vector representation. In the following, a short description of each feature is explained.

- Maximum: Let *M* be a set of values. Max(M) will result in $m_{i}$s.t. Then $m_{i}\in M$ is the maximum value of that set.
- Minimum: Similarly, Min(M) will result in $m_{i}$s.t. Then $m_{i}\in M$ is the minimum value of the set.
- Average: Suppose a dataset *M* comprises *n* numbers, the average can then be computed as:
  (1) $$Average\left(M\right)=\mu =\frac{\sum_{i=1}^{n}m_{i}}{n}$$
- Standard deviation: It is the average deviation of data points from the distribution center (i.e., μ) and can be calculated as:
  (2) $$StdDev\left(M\right)=\sigma =\sqrt{\frac{\sum_{i=1}^{n}{\left(m_{i}-\mu \right)}^{2}}{n-1}}$$
- Zero crossing: This feature determines how many times the specified set of values have zero crossings in the data [53]. Specifically, it can be considered a location on a mathematical function’s graph where the axes intercept, that is, when the graph crosses zero in either direction.
- Percentiles: This feature represents the number below a specific percentage of values in data. Specifically, a *q*th percentile would be a value in the dataset such that, at most, (100 × *q*)% of the data points fall below this value and 100 × (1 − *q*)% of the values fall above. That is, the 25th percentile (also known as the first quartile) reveals a feature whose value is greater than 25% and less than 75% in the dataset. Similarly, the 50th and 75th percentiles are represented by second and third quartiles, respectively. In the proposed technique, we computed three distinct percentiles features: (1) percentile 20, (2) percentile 50, and (3) percentile 80.
- Interquartile range: The first quartile value is subtracted from the third quartile value to obtain the interquartile range.
- Kurtosis: This feature is a measure to quantify the variations in the tails of a distribution from a normal distribution [54]. A large value represents a higher extremity of deviation, i.e., outliers. It can be formulated as:
  (3) $$Kurtosis=\frac{\sum {\left(m_{i}-\mu \right)}^{4}}{n\sigma^{4}}$$
- Skewness: Skewness describes the dataset’s divergence from the normal distribution. That is, it measures the asymmetry of normal distribution in either direction.
  (4) $$skewness\left(M\right)={\tilde{\mu }}_{3}=\frac{\sum_{i=1}^{n}{\left(m_{i}-\mu \right)}^{3}}{n\sigma^{3}}$$
- Auto-correlation: It is a numerical quantity to measure the similarity between the data at time $t_{1}$ and a lagged version of data in a temporal direction (at time $t_{2}$). Conceptually, it estimates the correlation between the current data and previous values [55]. It can be formulated as:
  (5) $$R_{k}=\frac{\sum_{i=1}^{n-k}\left(m_{i}-\mu \right)\left(m_{i+k}-\mu \right)}{\sum_{i=1}^{n}{\left(m_{i}-\mu \right)}^{2}}$$
- Order mean values: To compute order mean values, the data are arranged in an ascending order. The smallest value in the sorted dataset corresponds to the first-order statistic. The second-order statistic is the next smallest number, and so on. In the proposed technique, we computed four distinct features using order mean values: (1) First-Order Mean (FOM), (2) Norm of FOM, (3) Second-Order Mean (SOM), and (4) Norm of SOM. We employed two of the mostly used norm techniques: $L_{1}$ (i.e., Manhattan distance) and $L_{2}$ (i.e., Euclidean distance).
- Spectral entropy: The spectral entropy (SEP), which gauges a signal’s spectral power distribution, is based on the Shannon entropy idea. The time-based signal was transformed into its frequency spectrum using the Fourier transform. The standardized power distribution in the frequency domain is taken into account as a probability density function to calculate the signal’s Shannon entropy:
  (6) $$SEP=-\sum_{i=1}^{n}\hat{F}\left(n\right)\times log\hat{F}\left(n\right)$$
- Spectral energy: Since various sensory data can be used to assess walking patterns in the recorded data, they may be considered as a function whose amplitude varies with time. Similar to SEP, the time-based signal was transformed into its frequency spectrum using the Fourier transform, and the signal energy distribution over the frequency was calculated using the spectral energy formulation:
  (7) $$SE=\sum_{i=1}^{n}F{\left(n\right)}^{2}$$
  where F(n) is the amplitude of frequency content. It may be formulated using normalized frequency spectra:
  (8) $$\hat{F}\left(n\right)=\frac{F(n)}{\sum_{i=1}^{n}F\left(n\right)}$$
  All the abovementioned feature quantities were computed on each walk pattern and they were fused together in a single row to form a feature vector representation.

### 4.2. Codebook-Based Feature Encoding

**Figure 3:**
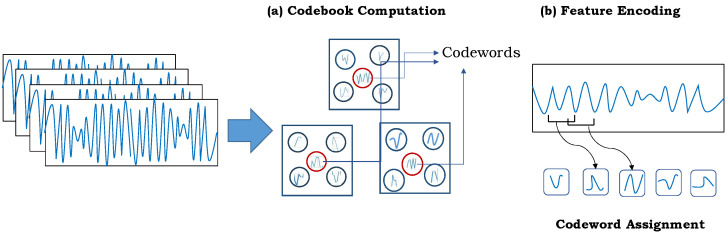
An illustration of codebook-based feature encoding technique.

The codebook-based feature encoding technique has shown excellent recognition results in numerous applications of gait recognition [9], image classification [56], and activity analysis [57]. It follows the BOVWs channel, which has two main steps: (1) codebook computation and (2) feature encoding. The complete process is illustrated in Figure 3, an and explanation of each task is outlined in the following.

#### 4.2.1. Codebook Computation

In the first step, the input data are distributed into groups based on their underlying similar patterns using a clustering algorithm to create a dictionary. Usually, the codebook is constructed using unlabeled data. Each of the cluster center in the dictionary is known as a codeword, which describes the underlying variability in the gait data. We divided the continuous time series sensory data into equal-length sub-sequences using a window-sliding approach. In particular, a window of size *w* is moved at every l−th sensory data point with a small overlap between neighboring sub-sequences. We grouped all the segmented sub-sequences into different *k* clusters using a k-means clustering algorithm. The clustering method was run ten times to choose the best clusters with the minimum sum of Euclidean distances between the data and the centers of their respective clusters. Assuming that {$x_{m},m=1,\ldots ,M\}$ are the sub-sequence, $c_{k}$ is the set of *k* clusters, and $r_{mk}\in \left\{0,\;1\right\}$, the distance is computed as:

(9) $$minJ\left(r_{mk},c_{k}\right)=\sum_{m=1}^{M}\sum_{k=1}^{K}r_{mk}{∥x_{m}-c_{k}∥}_{2}^{2}$$

That is, the objective in Equation (9) is to minimize the cost function J on $r_{mk}$ and $c_{k}$ values. The optimal number of clusters *k* is chosen empirically.

#### 4.2.2. Feature Encoding

This explains the process to encode the raw sensory data into its final representation, which is usually computed by estimating the distribution of these codewords in the gait input sequence. To this end, we employed locality-constrained linear coding (LLC) to encode the gait sub-sequences into a single high-level representation. LLC belongs to the category of reconstruction-based encoding techniques [12]. It emphasizes the decoding process’ characteristics such that the feature *v* is enforced in order to reassemble the sub-sequence *x* [16]. Rather than sparsity, LLC uses the locality constraint to map each gait sub-sequence *x* into its own local coordinate system. Specifically, it projects each sub-sequence *x* into its local coordinate system using the locality constraint [58]. This constraint can be applied by reducing the distance between *x* and the codes used to reconstruct it. In order to calculate the coding coefficient, an encoded vector *v* is generated for *x* such that the gait sub-sequence *x* is identical to the product of *C* and *v* (i.e., Cv). It thus solves the following optimization problem:

(10) $$v=arg\min_{v}{∥x-Cv∥}_{2}^{2}+\lambda {∥d⊙v∥}_{2}^{2},\;s.t.1^{M}v=1,$$

where *x* represents gait sub-sequence, $C=\{C_{k},k=1,\ldots ,K\}$ is codebook, λ is the regularizer parameter whose value is set to 0.01 [12], and ⊙ explains the member-wise product. The term $d_{}=\left(exp\left(dist\left(x,C\right)/\sigma \right)\right)$ in Equation (10) indicates the locality adaptor where σ is applied to adjust the speed of weight decay *d*. Additionally, the dist(x,C) can be computed as: ${∥x-c_{1}∥}_{2},{∥x-c_{2}∥}_{2},\ldots ,{∥x-c_{k}∥}_{2}$. Since *C* is the *k* closest cluster to sub-sequence *x*, a computational efficient LLC solution can be employed by selecting the *k*-nearest basis vectors of sub-sequence *x* to minimize the term ${∥x-c_{k}∥}_{2}^{2}$ in Equation (10). To this end, we set the value of *k* to 5 empirically.

### 4.3. Classification

In the framework of handcrafted features and BOVWs, the final step is the classification of gait sequences. We evaluated the performance of two well-known and mostly used machine learning algorithms for classification: Support Vector Machine (SVM) and Random Forest (RF). In the following, a brief summary of each classification tool is outlined.

#### 4.3.1. Support Vector Machine (SVM)

We employed simple linear SVM implementation to recognize the aforementioned computed feature representation. SVM has been used as a classification tool in numerous recognition applications [57]. SVM follows the principle of margin maximization; it learns a decision boundary (e.g., hyperplane) between the different classes. The data points closest to the hyperplane are known as support vectors and have the greatest influence on the adjustment of the hyperplane’s location. SVM learns the following optimization problem to learn an optimal hyperplane:

(11) $$\begin{array}{c} \min_{w}\;\frac{1}{2}{∥w∥}^{2}+C\sum_{i=1}^{N}\xi_{i}\\ s.t.\;y_{i}\left(w^{T}x_{i}+b\right)\geq 1-\xi_{i},\;\;\xi_{i}\geq 0,\;\;\forall i \end{array}$$

where *w* is the weight vector, C>0 is a penalty to misclassification, and $\xi_{i}=max{\left(0,1-y_{i}w^{T}x_{i}\right)}^{2}$ is a loss function. The term $y_{i}=\left\{-1,+1\right\}$ is the label associated with training instance $x_{i}$. The objective of the SVM training is margin maximization, which is approximately equal to minimizing the regularization term ${∥w∥}^{2}$. To optimally select the hyperparameter *C*, a 10-fold cross-validation is performed. The proposed technique employed the implementation of the LIBLINEAR SVM library [59]. For multi-class classification, it employed the one-vs.-rest strategy.

#### 4.3.2. Random Forest (RF)

**Figure 4:**
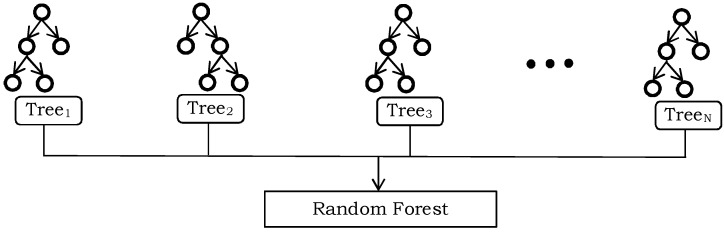
An illustration of the Random Forest classifier.

We also evaluated the effectiveness of RF [60] to recognize the high-level feature representation of gait sequences. RF has also been proven to be an efficient classification tool which can handle multi-dimensional features. It does not require any cross-validation, runs efficiently on large datasets, and is capable to work with missing data (to some extent). It is based on the principle of decision trees and generates ensemble learning, which involves a lot of classification trees to solve complex problems. For classification, the computed feature representation is passed in each of the forest’s tree. At the end of every tree, it produces a classification score, known as “vote”. The predicted classification with more votes is the object class. The complete process is illustrated in Figure 4. The classification accuracy of Random Forest depends on the correlation of any two trees and the strength of each individual tree.

### 4.4. Deep Learning-Based Feature Extraction Techniques

In recent studies, deep learning-based automatic feature extraction techniques have been explored to analyze gait patterns due to their robustness and efficient feature extraction, generalization power, and convincing recognition accuracies. These techniques present an end-to-end deep architecture to learn high-level characteristics from raw sensory data using a fully automated feature extraction process. They compute a high-level representation of input data using the complex structure of the deep artificial neural networks. In this study, we presented two end-to-end deep networks for gait analysis and activity recognition: (1) convolutional neural network (CNN) and (2) long short-term memory (LSTM). Each technique is briefly described in the following subsections.

#### 4.4.1. Convolutional Neural Network (CNN)

A CNN is a deep network to learn high-level features directly from input data. Although they are primarily designed to extract the high-level semantics in images or videos to recognize objects, they are also proven to be effective in the classification of audio and time series data. A CNN usually consists of several layers of artificial neurons, which are mathematical functions that calculate the weighted sum of multiple inputs and output an activation value (similar to biological neurons). Each layer of CNN accepts input from the previous layer, computes a specific feature from it, and forwards it to the following layer as input [61].

Typically, a CNN consists of three different types of layers: (1) convolutional, (2) pooling, and (3) fully connected layers. The convolutional layer is considered an essential block of CNN which performs convolutional operations using a certain number of filters with their specified sizes. It convolves the filters on input data, extracts the useful features from raw data, and passes the result to the next layer. The pooling layer plays a vital role in reducing the dimensionality of input data by eliminating the number of feature points in the output from the previous convolution layer. Specifically, the convolutional layer identifies some important region of the input data, whereas the pooling layer conceals its exact location and keeps the most important information. Finally, a fully connected layer maps the extracted features to the final output, such as classification [62].

**Figure 5:**
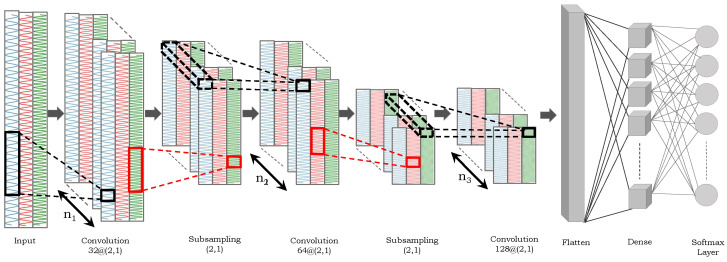
The proposed deep CNN consists of three convolutional, two sub-sampling, one flattened, and one dense layer.

The proposed CNN follows the sequential layered approach with inputs as raw sensory sequences. The network configuration is shown in Figure 5. More specifically, it consists of three convolutional, two sub-sampling, one flattened, and one dense layer. The input layer is followed by three Conv2D layers with 32, 64, and 128 filters, respectively. Each Conv2D layer uses a kernel size of (3, 2), (2, 1), and (2, 1), respectively, and a ReLU activation function. Two MaxPooling2D layers follow the first and second Conv2D layers with a pool size of (2, 1), which reduces the spatial dimensions of the feature maps. The third Conv2D layer is followed by a flattened layer. The output from the preceding layer is flattened in this layer into a one-dimensional (1D) vector that can be fed into a dense layer. Finally, a probability distribution across the available classes is produced by the dense layer with Softmax activation function. The number of epochs is 27 with a batch size of 5.

#### 4.4.2. Long Short-Term Memory (LSTM)

The LSTM is a type of recurrent neural network (RNN) which is designed to learn long-term dependencies, particularly in sequence prediction scenarios of time series data. LSTM differs from other neural networks mainly due to the way it deals with information over time. The data processing method used by traditional deep networks is feed-forward. Contrarily, LSTM-based deep networks process data in a recurrent manner. That is, they accept input data at one timestamp and produce an output at the following timestamp. A typical RNN is unable to learn longer-term dependencies due to the “vanishing gradient” or “exploding gradient” problem in back-propagation training. Contrarily, LSTM-based deep networks use additional gates to limit this issue. These gates enable the network to learn longer-term dependencies by controlling what the information in the hidden cell is exported as output and to the next hidden state. LSTM-based deep networks employ feedback connections to process the entire data sequence and have shown convincing recognition results in the domain of speech recognition, activity classification, etc.

**Figure 6:**
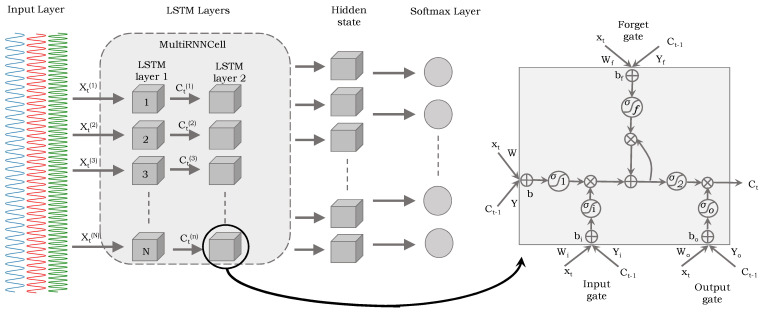
An illustration of the proposed LSTM-based deep network to classify the multimodal sensory data.

An LSTM architecture consists of four main components: (1) memory cell, (2) input gate, (3) forget gate, and (4) output gate. The memory cell is the memory of the network which is used to memorize the information from an earlier timestamp and the current one. The input gate controls how the cell state is updated with the information from the current timestamp. The output gate regulates what information is output from the cell state at the current timestamp, while the forget gate controls what information from the previous timestamp is maintained in the present timestamp, as depicted in Figure 6. All these gates work on input *x* at timestamp *t* (i.e., $x_{t}$) and the $Y_{t-1}$ by following the equations:

$$f_{t}=\sigma \left(W_{f}\cdot \left[Y_{t-1},x_{t}\right]+b_{f}\right)$$

$$i_{t}=\sigma \left(W_{f}\cdot \left[Y_{t-1},x_{t}\right]+b_{i}\right)$$

$${\tilde{C}}_{t}=tanh\left(W_{C}\cdot \left[Y_{t-1},x_{t}\right]+b_{C}\right)$$

$$C_{t}=f_{t}⊗C_{t-1}+i_{t}⊗{\tilde{C}}_{t}$$

$$o_{t}=\sigma \left(W_{o}\cdot \left[Y_{t-1},x_{t}\right]+b_{o}\right)$$

$$h_{t}=o_{t}⊗tanh\left(C_{t}\right)$$

where $x_{t}$ represents the input of the current timestamp, $Y_{t-1}$ denotes the hidden state of previous timestamp, *W* is the weight matrix associated with the hidden state, *b* are the bias vectors for gates, $C_{t}$ is the state of the cell at time *t*, σ indicates the activation functions to squeeze the input and output of the cell, and ⊗ represents the point-wise multiplication operations. The initial part determines whether the information from the earlier timestamp should be remembered or (if it is unnecessary and) can be ignored. Using a sigmoid function, the forget gate determines which data from the previous cell state need to be forgotten. It considers the values of $Y_{t-1}$ and $x_{t}$, and generates a value between 0 and 1. The cell attempts to learn new knowledge in the second section using the input provided to it. With the help of point-wise multiplication operations of “sigmoid” and “tanh”, the input gate regulates the information flow to the present cell state.

First, the input gate layer selects the values that will be updated. Next, a vector of potential new values ${\tilde{C}}_{t}$ (that could be added to the state) is created using a tanh layer. Later, these two values are combined to update the cell state. The equation of ${\tilde{C}}_{t}=tanh\left(W_{C}\cdot \left[Y_{t-1},x_{t}\right]+b_{C}\right)$ will be used to update the old cell state $C_{t-1}$ into the new cell state $C_{t}$. Specifically, the old state is multiplied with $f_{t}$ to forget, and the product of $i_{t}$ with ${\tilde{C}}_{t}$ is added. This is the updated value, scaled by the amount by which we decided to alter each state value. Finally, the output gate determines which data should be sent to the following hidden state. Similarly to a simple neural network, the LSTM cells are organized in layers. The output of the cell in each layer is forwarded to the cell in the next successive layer. Finally, dense and Softmax layers are used in the network to accept the output of the last layer for recognition.

## 5. Experiments and Results

This paper mainly emphasizes on a systematic evaluation of numerous feature learning techniques to encode multimodal time series sensory data for gait analysis. However, the proposed feature encoding techniques were also explored to determine whether they could recognize human daily activities, which were also recorded using sensory data. Therefore, we explored several datasets to assess the proposed features on both gait and daily activities.

### 5.1. Dataset Description

**Figure 7:**
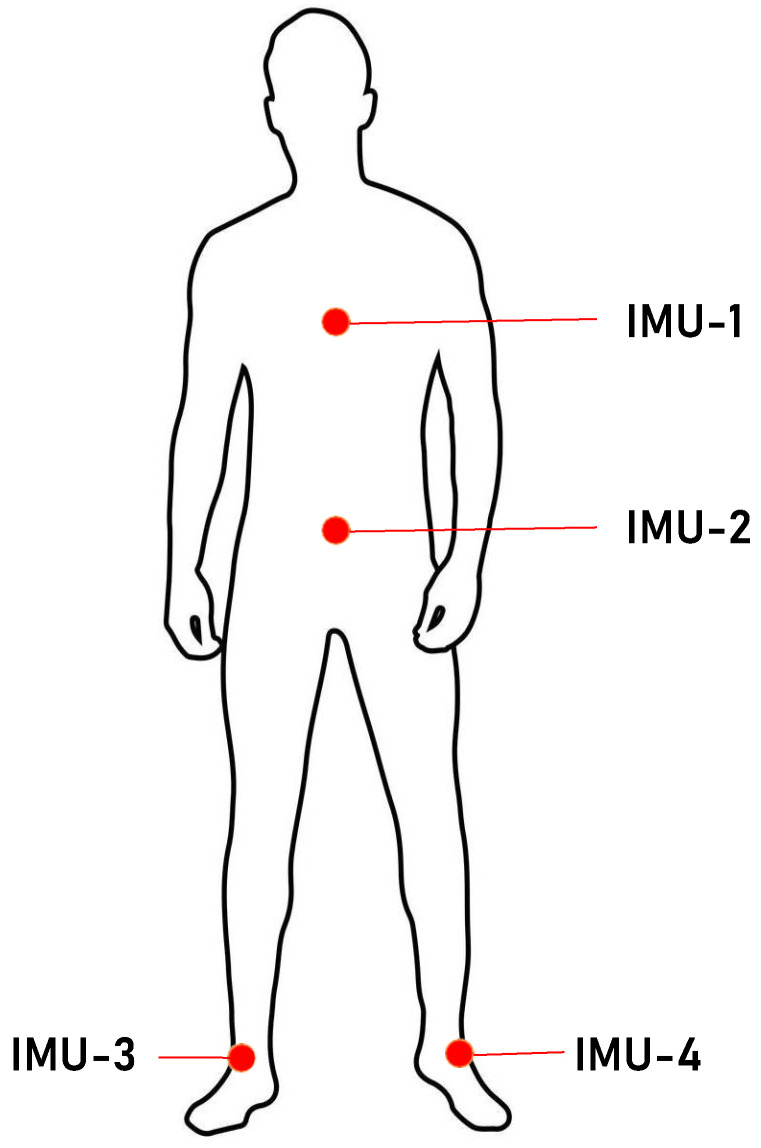
An illustration of IMU placement in collected dataset to capture the motion information of different body parts.

The proposed feature encoding techniques are evaluated on four large sensory datasets, and their details are briefly explained in the following:

- Gait-IMU dataset: The first dataset is collected in our APPS lab, located at the University of Lübeck, Germany. The IMU data are collected using LPMS-B2 Series (Advanced Realtime Tracking GmbH & Co. KG, Weilheim i.OB, Germany) devices (https://www.lp-research.com, accessed on 20 August 2020) along with the SensFloor sensor to analyze the gait patterns. A preliminary study on the analysis of SensFloor sensory data for gait analysis has been published in [63]; however, IMU data were not explored. Since an IMU consists of several sensors (e.g., accelerometer, gyroscope, magnetometer), the collected multimodal sensory data can be used to analyze gait in a more effective way. We sampled the IMU data at a fixed rate of 50 Hz. A total of 42 individuals participated in the gait collection. The four IMUs were attached to different body parts as depicted in Figure 7. The first IMU was attached at the sternum (chest), the second was at the lower abdomen (belt buckle), the third was at the left ankle, and the forth was at the right ankle. The participants performed six different walking styles: normal, slow, fast, blindfolded, dual task, and post-UHR by walking back and forth 10 times. The SensFloor data were used to identify the turning locations, and from there, a cut mark was placed to indicate the start and stop times of a single trip. More detail about the SensFloor data can be found in [63]. During data collection, we also observed a few missing data points (i.e., NAN values) in recorded data perhaps due to sensor malfunctions, which were covered using interpolation. However, we dropped the sequences having more than 30% NAN data points in the recorded sequences. This pre-processing makes the raw data suitable to be used with the feature encoding techniques. A total of 238 cleaned gait sequences are saved and freely available to the research community at https://drive.google.com/drive/folders/15sQTn3P2x3M1Em5o8yz1U784tomXDsJW (accessed on 20 August 2020).
- MHEALTH dataset: The second dataset we used to assess the effectiveness of the proposed feature encoding techniques is the MHEALTH (Mobile Health) dataset [17]. The dataset contains data on the body motions of ten volunteers of diverse profiles engaged in twelve different physical activities (e.g., walking, jogging, running, jumping, etc.). The recordings were made in a laboratory using four wearable sensors from Shimmer2 (https://shimmersensing.com, accessed on 20 August 2020). The sensors were attached on the chest, right wrist, and left ankle using elastic bands. The authors captured the dataset at a fixed sample rate of 50 Hz for all the modalities. These sensors capture the motion information of the human body in different aspects, namely acceleration, magnetic field orientation, and the rate of turn, which can effectively record the dynamics of the body. This dataset is quite useful to not only analyze the walking styles, but to also recognize the subjects’ daily activities.
- WISDM-AR dataset: The third dataset we used in this study was the Wireless Sensor Data Mining (WISDM-AR) dataset [18]. The data of twenty-nine volunteers were collected using a smartphone. While performing different daily activities, the subjects were instructed to carry their Android smartphones in their front leg pockets. The subjects performed different sets of activities, including walking, jogging, ascend and descend stairs, etc., for a certain period of time. The authors captured the dataset at a fixed sample rate of 20 Hz for all the modalities. Similar to the MHEALTH dataset, this dataset is also used to analyze the walking styles and the subjects’ daily activities.
- UCI-HAR dataset: This dataset comprises the sequences of activities of daily living [19]. The data were collected using a Samsung Galaxy S II sensor, which was attached to the waist of the participants during recording. Specifically, the data from the smartphone’s gyroscope and accelerometer sensors were collected at a frequency of 50 Hz. The dataset consists of a total of 748,406 sample points from 30 participants ranging in age from 19 to 48 years. The activity sequences are grouped into six classes, namely sitting, standing, walking, walking downstairs, walking upstairs, and lying down.

We performed different experiments to assess the performance of the proposed approaches. Similar to [64,65,66,67], we employed an overlapped and fixed-size sliding-window approach in all the experiments. The window size is set to 128 with a 50 percent overlap (i.e., 128 data points/window is segmented) to compute the features. Details of the different experiments on each of the dataset are explained below.

### 5.2. Analysis of Gait-IMU Dataset

The time series data of each walk were extracted using all the four IMU sensors. The recorded gait sequences contain three-dimensional (3D) data for each sensor, i.e., accelerometer, magnetometer, and gyroscope. Due to limited instances in the dataset, we employed only handcrafted and codebook-based feature encoding techniques. It is important to mention that the proposed techniques were evaluated on the data of each IMU sensor to assess their effectiveness individually.

#### 5.2.1. Using Handcrafted Features

We extracted eighteen handcrafted features (Section 4.1) from each walking sequence and they were fused together using feature-level fusion [57], i.e., through simple concatenation. The features were computed from each of the sensor, i.e., accelerometer, gyroscope, and magnetometer. Since each of the sensor generates 3D data, the final length of the feature vector was 3×3×18. The feature vectors were divided into training and testing sets with the ratio of 80 and 20, respectively. We trained the classifiers (SVM and RF) using training instances, and the testing sequences were fed to classifiers. The recognition results are summarized in Table 1. It can be shown that the proposed approach achieved a 100% recognition rate on multiple IMUs, which reveals the effectiveness of the proposed feature encoding technique. We further concluded that the best outcomes can be obtained even with just one IMU on an ankle.

#### 5.2.2. Using Codebook Approach

We computed a codebook for each type of sensory datum using its 3D sub-sequences. In particular, we segmented a sub-sequence with a window of size *w*, representing a 3w-dimensional vector. That is, the first, second, and third *w* represents the *x*, *y*, and *z* axes of sensory data, respectively. The intuitive reason behind this segmentation approach is to capture the correlation among three axes. The same sub-sequence segmentation approach was used in the encoding of the final feature vector. We empirically set the value of *w* to 64, and *l* to 8. It can be noted that we set a large overlapping with a small size of *l*, considering the fact that a sufficient number of sub-sequences can be collected to capture the temporal correlation in the sub-sequences. The number of clusters in codebook (i.e., k) was empirically set to 32. The high-level feature representation of the segmented sub-sequences was obtained using LLC encoding technique (Section 4.2.2) and it was fed to classifiers (SVM and RF) for walking style recognition. The recognition results are summarized in Table 1.

It can be observed from the recognition results that handcrafted features using RF as classifier performs consistently better than the codebook-based features. Since the dataset consists of only a few gait sequences, we cannot evaluate the deep learning-based techniques on this dataset.

### 5.3. Analysis of MHEALTH Dataset

We performed three different types of experiments on the MHEALTH dataset to evaluate the performance of the proposed techniques. Following the recommendation in [17], all the IMU sensor’s data were fused together (i.e., descriptor level fusion [11]) and used as input to feature learning algorithms. Details of the different experiments on each of the dataset are explained in the following.

#### 5.3.1. Using Handcrafted Features

To compute the handcrafted features, we followed the recommendation in [17] and used the raw data of all the sensors with a window size of 20×23. To capture the body dynamics comprehensively, the subject’s chest, right wrist, and left ankle were equipped with sensors using elastic straps. By employing multiple sensors, we were able to measure various aspects of motion, including acceleration, rate of turn, and magnetic field orientation. Therefore, they jointly provided a more comprehensive understanding of the body’s movement. All the extracted handcrafted features were fused together, indicating the final representation of the walk or activity. Finally, the features were fed to a classifier for recognition. The recognition results are summarized in Table 2. It can be noticed that the proposed handcrafted feature achieved the highest recognition rate of 99% using RF as classifier.

#### 5.3.2. Using Codebook Approach

Similar to the Gait-IMU dataset, we computed a codebook using a k-means clustering algorithm, and the high-level feature representation was obtained using an LLC encoding technique. We recall that all the IMU sensor’s data were fused and used together as input for codebook computation and feature encoding. A recognition rate of 85% was achieved, as depicted in Table 2.

#### 5.3.3. Using Deep Learning Approaches

We recall that two end-to-end deep learning models are presented to automatically extract features from raw sensory data. In the following, the implementation details of each model are presented.

CNN: We recall that the proposed 2D-CNN consists of one input layer, three convolution layers, two sub-sampling layers, one flattened layer, and one dense layer. The first convolution layer used 32 filters of size 3×2, the second layer used 64 filters of size 2×1, and the third layer used 128 filters of size 2×1. For each sub-sampling layer, the stride factor was set to 2×1 to reduce the spatial dimensionality of the data. The convolution layer’s bias term was set to true, the number of epochs was 27 with a batch size of 5, and Rectified Linear Unit (ReLU) was used as an activation function. The network is build using Keras framework [68] with tensor flow as back end [69].

To determine the best values for the hyperparameters of the presented deep network (e.g., the number of layers, the activation function, etc.), we employed a multi-resolution search strategy [70]. In particular, it chooses parameter values from a wider range in the first phase and selects a few optimum configurations. The best values are then chosen from a small search space near these values. For instance, we evaluated the performance of the proposed deep network in a an increasing number of layers (conv2D and MaxPooling2D) and stopped when the performance on the validation data reached at its peak. It was empirically concluded that increasing the number of layers beyond five did not improve the performance. We used the EarlyStopping callback mechanism to monitor the validation loss, and the best weights were learned. We achieved a 99% recognition rate on MHEALTH dataset, and the results are summarized in Table 2.

LSTM: The proposed LSTM-based deep network consists of four LSTM layers, one flattened layer, and one dense layer. The model accepts an input tensor X with the shapes of [batch_size, segment_time_size, no_of_features], which were set to 32, 200, and 3, respectively. The model starts by initializing the weight *W* and bias *b* of the hidden layer and output layer using random normal distributions. The input tensor X is then transposed and reshaped to a 2D tensor of size (batch_size * segment_time_size, no_of_features), which is fed to the hidden layer. The hidden layer applies the ReLU activation function to the input tensor, and then splits the output into segment_time_size tensors.

Similar to CNN, the network is built using Keras framework [68] with tensor flow as back end [69]. We stacked two LSTM cells on top of each other to form a multi-layered LSTM. Specifically, the tf.contrib.rnn.BasicLSTMCell function is used to create each LSTM cell with a hidden state size of 50 neurons and a forget bias term of 1.0. The tf.contrib.rnn.MultiRNNCell function is used to create a multi-layered LSTM using two LSTM cells. The tf.contrib.rnn.static_rnn function is used to run the LSTM model over the input sequence. The output for the last time step of the LSTM is extracted from the output’s tensor using indexing and fed to the output layer. The output layer applies a linear transformation to the input tensor, and the final output is obtained by multiplying the result with *W* and adding b. A multi-resolution search [70] approach is used to learn the optimal values for the hyperparameters of the proposed deep network (i.e., number of layers, activation function, etc.). We used the EarlyStopping callback mechanism to monitor the validation loss, and the best weights were learned. We achieved a 96.78% recognition rate on MHEALTH dataset, and the results are summarized in Table 2.

The performances of the proposed feature encoding techniques are also assessed with the existing state-of-the-art techniques and their accuracies are outlined in Table 3. The recognition results reveal that the proposed feature encoding techniques outperform the existing state-of-the-art techniques.

### 5.4. Analysis of WISDM-AR Dataset

The third dataset we used to assess the effectiveness of the proposed feature encoding techniques is the WISDM-AR dataset [17]. A short description of each of the feature encoding techniques is summarized below.

#### 5.4.1. Using Handcrafted Features

Following the recommendation in [18], the subsets of each activity are used to extract the handcrafted features in order to make a fair comparison. We computed the aforementioned eighteen handcrafted features and they were fed to a classifier (after fusion) for recognition. The recognition results are summarized in Table 2. It can be observed that the proposed handcrafted feature encoidng tehcnique achieved a recognition rate of 94%.

#### 5.4.2. Using Codebook Approach

We computed the codebook using a k-means clustering algorithm for the WISDM-AR dataset, and the activity sequences were transformed to high-level descriptors using the LLC encoding technique. It achieved a recognition rate of 79%, as depicted in Table 2.

#### 5.4.3. Using Deep Learning Approaches

We employed the same implementation of CNN- and LSTM-based deep networks as explained in Section 5.3.3. The CNN achieved a recognition rate of 97%, whereas LSTM achieved a recognition rate of 94%.

The performance of the proposed feature encoding technique is also compared with the existing state-of-the-art techniques and their recognition scores are outlined in Table 4. The proposed feature encoding technique outperforms the current state-of-the-art techniques.

### 5.5. Analysis of UCI-HAR Dataset

The forth dataset we used to assess the effectiveness of the proposed feature encoding techniques is the UCI-HAR dataset [19]. We employed the leave-one-subject-out cross-validation technique to validate the generalization power of the proposed techniques. A short description of each feature encoding technique is presented below.

#### 5.5.1. Using Handcrafted Features

Similar to previous experiments, we extracted eighteen handcrafted features from the raw sensory data. All the features are concatenated to form a vector representation and fed to classifier for recognition. The recognition results are summarized in Table 2. It can be observed that the proposed handcrafted feature encoding technique achieved a recognition rate of 95.9%.

#### 5.5.2. Using Codebook Approach

We computed the codebook using a k-means clustering algorithm for the UCI-HAR dataset, and the sequences were transformed to high-level descriptors using the LLC encoding technique. It achieved a recognition rate of 92.3%, as depicted in Table 2.

#### 5.5.3. Using Deep Learning Approaches

We employed the same implementation of CNN- and LSTM-based deep networks as explained in Section 5.3.3. The CNN achieved a recognition rate of 96%, whereas LSTM achieved a recognition rate of 94%.

The performance of the proposed feature encoding technique is also compared with the existing state-of-the-art techniques and their recognition scores are outlined in Table 5. The proposed feature encoding technique outperforms the current state-of-the-art techniques.

### 5.6. Discussion and Computational Analysis

**Figure 8:**
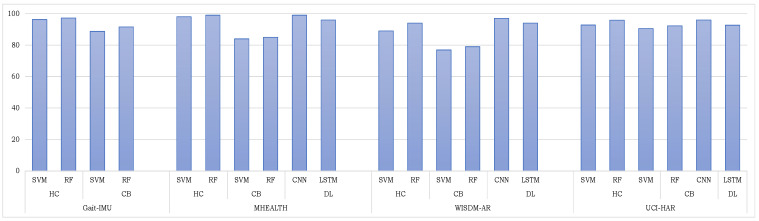
A summary of the recognition results of four datasets using all the encoding methods. The terms HC, CB, DL, SVM, RF, CNN, and LSTM represent handcrafted features, codebook features, deep learning features, Support Vector Machine, Random Forest, convolutional neural network, and long short-term memory, respectively.

The main aim of this research is to present a comparative study of several feature extraction approaches to encode multimodal time series data for gait analysis. To fulfill this task, we gathered the gait sequences of 42 individuals in our APPS lab (located at the University of Lübeck, Germany) using IMU sensor LPMS-B2 Series devices. We presented three feature learning techniques to encode multimodal time series sensory data for gait analysis. Among them, the proposed handcrafted feature extraction technique demonstrated excellent recognition results on the collected dataset. To further verify the robustness of the proposed feature encoding techniques, we performed another experiment to recognize the activities of daily living. We assessed the performance of the proposed techniques on four large activity datasets that are captured using different sensing modalities. A short description of different sensing modalities in each of the dataset is summarized in Table 6, and their recognition results are depicted in Figure 8. The results demonstrate that the proposed feature encoding techniques can encode the raw data of different sensing modalities. It establishes a solid foundation and generic framework to encode any time series multimodal sensory data. We empirically concluded that the combination of accelerometer, gyroscope, and magnetometer work well together to recognize human activities. The recognition scores of different feature encoding techniques in Table 1 and Table 2 have confirmed that handcrafted feature encoding technique using RF and CNN-based feature learning techniques outperformed the other remaining techniques. Furthermore, they also prove to be superior in comparison with existing state-of-the-art techniques, as shown in Table 3, Table 4 and Table 5. The excellent results demonstrate the applicability of the proposed techniques to work with different modalities. We believe that the proposed feature encoding techniques establish a solid foundation and generic framework to encode any time series multimodal sensory data. That is, it is capable to work with different sensing modalities such as SensFloor, skin temperature sensors, and electrocardiograms (ECGs); however, the classification of such data is beyond the scope of this research and can be explored in future work.

It is worth mentioning that the proposed technique is computationally inexpensive and does not require any expensive specialized hardware (e.g., GPU) for execution. All the experiments are performed on a machine with an Intel i5-4310U CPU with 16 GB RAM (Dell, Siegen, Germany). We also analyzed the computational complexity of different feature encoding techniques. Specifically, the average time for the encoding and classification of one activity/gait sequence is recorded. The results are reported in Table 7. The deep CNN- and LTSM-based features require an average time of 12.93 and 11.95 milliseconds (ms), respectively. The codebook-based feature encoding techniques seem to be more efficient and have an average of 10.5 ms for both feature encoding and classification; however, they require the prior computation of a codebook (once), which is quite a complex process, and require an additional 7.1 min to compute it. The proposed handcrafted feature encoding techniques require an average of 13 ms for feature encoding and classification, and they do not require any prior computation. It can be observed that the proposed techniques can classify the activity sequences almost in real time without any need for an expensive GPU. Among the three proposed feature encoding techniques, the handcrafted feature representation is computationally efficient; however, these features lack exploratory capabilities, rely on the domain knowledge of the researchers, and are application dependent [81]. It is quite difficult to build a generic framework based on these features. Additionally, they may not be scalable when used with different datasets and might show partiality toward certain features. The deep learning-based approaches perform poorly and suffer with overfitting (and underfitting) due to the limited number of instances in the dataset.

## 6. Conclusions

This paper presents three different feature encoding techniques to recognize different human walking styles using multimodal time series sensory data. The first technique extracts eighteen handcrafted features directly from the raw sensory data. The second technique follows the channel of Bag-of-Visual-Words (BOVWs) approach. The feature representation of sensory data is obtained using codebook- and LLC-based feature encoding techniques. For both of these feature representations, the performance of two different machine learning algorithms is assessed. In the third feature encoding technique, two different end-to-end deep learning models are presented to automatically extract the features from raw sensory data. An extensive experimental evaluation of all the feature encoding techniques is carried out on four large sensory datasets and their recognitions results are compared. The experimental evaluation reveals that the proposed features are quite robust and can be used to recognize human daily activities as well. A comparison of the proposed encoded feature with the existing state-of-the-art techniques reveals its superiority and effectiveness. This research also collected a large gait dataset using IMU sensors that we have made available to the research community. In future, we plan to explore more deep learning models (especially hybrid models) to automatically extract the features from raw sensory data.

## Figures and Tables

**Table 1:** Recognition accuracies (%) of all IMU sensors with eighteen handcrafted features and codebook-based features. The terms SVM and RF represent Support Vector Machine and Random Forest, respectively. The best results are marked in bold.

IMUs	Handcrafted Features		Codebook-Based Features	
	**SVM**	**RF**	**SVM**	**RF**
IMU-1	**92.6**	**92.6**	81.5	88.9
IMU-2	96.3	**100.0**	88.9	96.3
IMU-3	**96.3**	**96.3**	92.6	85.2
IMU-4	**100.0**	**100.0**	91.7	95.8
Average	96.3	**97.2**	88.7	**91.6**

**Table 2:** Recognition accuracies (%) of different feature encoding techniques on MHEALTH and WISDM-AR datasets. The terms SVM, RF, CNN, and LSTM represent Support Vector Machine, Random Forest, convolutional neural network, and long short-term memory, respectively. The best results are marked in bold.

	Handcrafted		Codebook		Deep Learning	
	**SVM**	**RF**	**SVM**	**RF**	**CNN**	**LSTM**
MHEALTH dataset	98.0	**99.0**	84.0	85.0	**99.0**	96.0
WISDM-AR dataset	89.0	94.0	77.0	79.0	**97.0**	94.0
UCI-HAR dataset	92.8	95.9	90.5	92.3	**96.0**	92.7

**Table 3:** Comparison of recognition results (%) of the proposed technique with the state-of-the-art techniques on MHEALTH dataset. The best results are marked in bold.

Methods	Year	Accuracy
Halloran et al. [71]	2019	83
Khatun et al. [72]	2022	93
Davidashvilly et al. [73]	2022	87
Yatbaz et al. [74]	2021	97
Nematallahet al. [65]	2020	93
Proposed handcrafted features with RF	2023	**99**
Proposed deep CNN	2023	**99**

**Table 4:** Comparison of recognition results (%) of the proposed technique with the state-of-the-art techniques on WISDM dataset. The best results are marked in bold.

Methods	Year	Accuracy
Xia et al. [64]	2020	95
Semwal et al. [75]	2022	90
Challa et al. [67]	2022	96
Xu et al. [76]	2018	91
Yin et al. [66]	2022	96
Proposed deep CNN	2023	**97**

**Table 5:** Comparison of recognition results (%) of the proposed technique with the state-of-the-art techniques on UCI-HAR dataset. The best results are marked in bold.

Methods	Year	Accuracy
Khan et al. [77]	2021	95.4
Xia et al. [64]	2020	95.8
Tong et al. [78]	2022	95.4
Perez et al. [79]	2021	94.7
Kolkar et al. [80]	2021	93.1
Proposed handcrafted features	2023	95.9
Proposed deep CNN	2023	**96.0**

**Table 6:** A description of the different sensing modalities that were used to gather the datasets and the obtained recognition results.

Dataset	Data Collection	Collection Rate	Sensing Modalities	Accuracy
Gait-IMU	The gait data are collected using IMU sensor LPMS-B2 Series devices.	50 Hz	Accelerometer, magnetometer, and gyroscope	97.2
MHEALTH	The activity data are collected using wearable IMU sensors from Shimmer2.	50 Hz	Accelerometer, magnetometer, and gyroscope	99.0
WISDM-AR	The activity data are collected using a smartphone.	20 Hz	Accelerometer	97.0
UCI-HAR	The activity data are collected using a smartphone.	50 Hz	Accelerometer, and gyroscope	96.0

**Table 7:** Analysis of computational time in feature encoding and classification. The average computation time for each of the encoding techniques is presented. The average feature encoding and classification time is reported for a gait/activity sequence on a CPU machine. The time is computed in milliseconds (ms).

	Feature Encoding (ms)	Classification Time (ms)
Handcrafted using SVM	5.07	8.98
Handcrafted using RF	5.07	9.44
Codebook using SVM	5.94	4.51
Codebook using RF	5.94	4.51
CNN	-	12.93
LSTM	-	11.95

## Data Availability

The dataset of 238 gait sequences are freely available to the research community at https://drive.google.com/drive/folders/15sQTn3P2x3M1Em5o8yz1U784tomXDsJW (accessed on 14 October 2023).
